# Effects of low-level lead exposure on blood pressure and function of the rat isolated heart

**DOI:** 10.4103/0253-7613.41041

**Published:** 2008

**Authors:** Badalzadeh Reza, Norouzzadeh Ali, Heydari Azhdar, Asgari Alireza, Khoshbaten Ali

**Affiliations:** 1 Department of Physiology and Biophysics, Medical Faculty, Baqiyatallah University of Medical Sciences, Tehran-Iran; 2 Research Center for Chemical Injuries (RCCI), Baqiyatallah University of Medical Sciences, Tehran-Iran; 3 Department of Physiology, Medical Faculty, Tabriz University of Medical Sciences, Tabriz-Iran

**Keywords:** Cardiac contractility, ECG, hypertension, lead acetate

## Abstract

**Objective:**

Exposure to low levels of lead acetate can induce hypertension in both humans and experimental animals. The exact mechanisms of lead-induced hypertension are not well understood, but its pathogenesis could be explained by the changes in heart rate and contractility.

**Materials and Methods:**

In the present study, the effects of exposure to 100 ppm of lead in drinking water (for periods of 4, 8, and 12 weeks) on blood pressure and some physiologic parameters (eg, electrocardiography [ECG], heart rate [HR], cardiac contractility, and coronary flow) of isolated beating rat heart was investigated using the Langendorff isolated heart apparatus. The isolated hearts were perfused with Krebs-Henseleit solution (37°C; pH 7.4; gassed with 95% O_2_ + 5% CO_2_). All data were digitized by a software program for further analysis.

**Results:**

The blood pressure in the 8- and 12-week lead-exposed groups was significantly increased as compared to the control group. The ECG showed arrhythmias and conduction abnormalities only in the late phases of exposure (12 weeks). The HR and contractility were significantly higher in the 8- and 12-week lead-treated rats but not in the 4-week group. No significant changes were observed in coronary flow.

**Conclusion:**

These results indicate that: 1) low levels of lead exposure do not significantly affect the ECG in the early phase, 2) low levels of lead exposure causes ECG changes in the late phases of exposure, and 3) this level of lead exposure can increase HR and cardiac contractility but has no effect on coronary flow.

Lead is a heavy metal that is distributed widely in our environment as a consequence of its increasing use in industries.[1] It is an important environmental pollutant, exerting toxic effects on human health.[2] Lead intoxication may cause neurological, hematological, gastrointestinal, and cardiovascular dysfunction in humans and experimental animals.[3–5] Several epidemiological and experimental reports have documented that lead exposure is one factor contributing to cardiovascular impairment.[4,6] In recent decades, the relationship between blood lead levels and blood pressure (BP) has been extensively investigated and chronic exposure to low levels of lead has been shown to cause hypertension in both humans and animals.[7,9] Other reported cardiovascular changes following lead exposure include alteration in ECG, impaired cardiac conduction, increased incidence of arrhythmias as well as increased susceptibility to catecholamine-induced arrhythmias, and alteration in the force of cardiac contraction. All these changes are brought about by chronic exposure to relatively high levels of lead,[10,11] These findings were mainly derived from *in vivo* studies done on humans; *in vitro* experimental studies were few. Although some studies have tried to investigate the mechanisms of lead-induced hypertension, no consensus exists. Hypertension may occur following any alteration in the function of the systems that regulate blood pressure. The experimental findings suggest that lead acts at multiple sites within the cardiovascular, renal, and nervous systems.[4,12,13] There are few reports concerning the effects of exposure to low levels of lead on cardiac function. Most researchers in this field have used high doses of lead so their findings may not be comparable with ours. A few studies have investigated the effects of exposure to lower doses of lead (especially over short periods), but the results have not been consistent.[11,14,15] Thus, the aim of this study was to determine the subchronic effects of a low level of lead acetate (100 ppm) on BP, ECG (QRS, ST, R-R intervals, and arrhythmogenesis), HR, cardiac contractility, and coronary flow in male rat isolated heart.

## Materials and Methods

Male Sprague-Dawley rats (Pasteur Institute, Tehran-Iran) of 250-300 g body weight were used. The rats had free access to food and water and were housed at a temperature of 23 ± 2°C with a 12-h light/dark cycle. The animals were randomly divided into control and three lead-treated (4-, 8-, and 12-weeks) groups. Six to ten animals were allocated to each group.

### Exposure protocol

The three lead-treated groups were given drinking water containing 100 ppm (0.01%) of lead acetate for periods of 4, 8, and 12 weeks; control rats were given drinking water without any lead acetate.

### Chemicals

Lead acetate and all compounds used for making the Krebs-Henseleit solution were obtained from Merck Co., Germany.

### Blood pressure measurement

At the end of intoxication periods, the animals were anesthetized with a mixture of ketamine (75 mg/kg) and xylazine (25 mg/kg). The rat tail was placed inside the tail cuff, which was inflated and released a few times to allow the animal to be conditioned to the procedure. Systolic blood pressure values (three consecutive readings) were recorded using a tail sphygmomanometer (PE 300, Narco Bio-systems, USA) attached to a polygraph (MK III-P, Narco Bio-systems); the values were averaged for analysis.

### Determination of lead in blood

The lead content of whole blood was measured using an atomic absorption spectrophotometer (Shimadzu 680A, with graphite furnace; Shimadzu, Japan) and expressed as micrograms per deciliter (μg/dl).[9]

### Surgical procedure

After anesthesia and intraperitoneal injection of 500 IU heparin as anticoagulant, the animal thorax was opened from bilateral axillary's lines up to the first rib under artificial ventilation. Then, the ascending aorta was cannulated and the exposed heart was carefully isolated from the body (all the vessels were cut) and transferred immediately (within 30 s) to the Langendorff rat isolated heart apparatus. The isolated hearts were continuously perfused with Krebs-Henseleit solution (at 37°C, pH 7.4 and gassed with 95% O_2_ + 5% CO_2_); the solution had the following composition (in mmol/l): NaCl, 118; KCl, 7.4; NaHCO_3_, 25.0; KH_2_ PO_4_, 1.2; CaCl_2_, 2.5; MgSO_4_.7H_2_ O, 1.2; and glucose, 11.0. A 15-min period was allowed for the heart to reach a steady-state condition prior to any treatment.[16]

## Parameters Recorded

*ECG:* The ECG was recorded by three surface silver electrodes (2 active and 1 reference) placed on the surface of the isolated heart in the axis of lead II with a hi-gain coupler (Narco Bio-systems). The QRS, ST, and R-R intervals and arrhythmogenicity were assessed. The HR was calculated from the R-R intervals.

*Cardiac contractility:* A strain gauge was connected to the apex of the heart; the contractile signals were sent to an isotonic myograph transducer via an isotonic myograph detector (Narco Bio-systems) attached to the strain gauge.

*Coronary flow:* Coronary flow was measured using a drop counter transducer and its coupler (Narco Bio-systems). The number of drops from the right atrium was sensed by the transducer sensor.

Finally, all online data taken from the heart were saved and analyzed using a specific software (Long soft, version 1.1, Iran) program.

### Statistical analysis

All results were expressed as mean ± SEM. For the blood pressure data statistical differences were evaluated by the unpaired t-test; the one-way ANOVA followed by Tukey HSD test as *post hoc* test was used for the other parameters. A *P* value less than 0.05 was considered significant.

## Results

There was no significant difference in body weight between the control and lead-treated groups before and after the treatment period. The blood lead concentration of the lead-treated rats was significantly higher than that of the controls (26.84 ± 2.23 μg/dl *vs* < 2 μg/dl).

### Blood pressure

**Figure 1:**
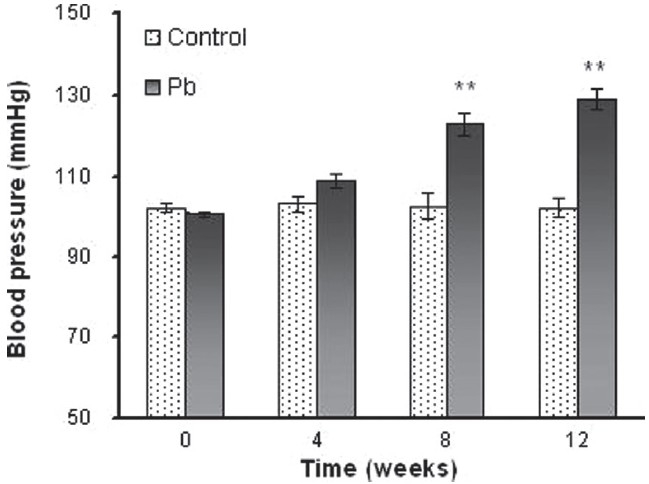
Blood pressure in control and in 100 ppm lead-treated (Pb) groups. Data presented as mean ± SEM (n = 6; **P < 0.01)

Systolic BP increased with increasing exposure periods from 0 to 12 weeks (100.68 ± 1.01 *vs* 129.18 ± 2.64 mmHg) [Figure 1]. The increase in BP in the 4-week lead-treated groups was not significant, but in the 8- and 12-week lead-treated rats there was significant increase in the BP as compared to their respective controls. This parameter remained unchanged in the control groups throughout the 12 weeks of the experiment.

## ECG

The differences in the QRS interval were not significant in any group [Table 1]. There was significant change in the ST interval in both the 8- and 12-week lead-treated animals (*P* < 0.05 and *P* < 0.01, respectively, as compared to controls) but not in the 4-week group [Table 1]. Increased HR (ie, decreased R-R intervals) was seen in both 8- and 12-week lead-treated groups (*P* < 0.01 and *P* < 0.05, respectively, as compared to controls) but there was no relationship between tachycardia and exposure period [Table 1].

**Table 1:** ECG parameters (HR (beat/min), QRS duration (ms) and ST interval (ms)), Cardiac contractile force (tension, in gr) and Coronary flow (CF, in ml/min) in control and lead-treated (Pb) groups

*Groups*	*ECG parameters*			*Tension_(gr)_*	*CF_(ml/min)_*
			
	*HR_(beat/min)_*	*QRS duration_(ms)_*	*ST interval_(ms)_*
Control	306.33 ± 10.62	4.67 ± 0.39	10 ± 0.75	3.29 ± 0.10	9.07 ± 0.41
4-week Pb	322.08 ± 8.94	4.93 ± 0.44	11.6 ± 1.23	3.52 ± 0.21	8.75 ± 0.37
8-week Pb	338.67 ± 11**	5.1 ±.038	13.72 ± 1.1*	4.14 ± 0.2**	8.62 ± 0.35
12-week Pb	329.83 ± 12.62*	5.38 ± 0.37	17.6 ± 2.51**	4.33 ± 0.11**	8.32 ± 0.30

Data presented as mean ± SEM, *n* = 10,

* *P* < 0.05,

** *P* < 0.01 (For presenting the HR in 12-week lead-treated groups, 4 rats with signs of arrhythmias and AV block were excluded and HR data of the other 6 rats were calculated and averaged for presentation)

Arrhythmias and signs of first and second degree atrioventricular (AV) conduction block were observed in the ECGs of 40% of the 12-week lead-treated rats; none of these findings were seen in the ECGs of the other three groups.

## Cardiac Contractility and Coronary Flow

At the end of the experimental periods, there was significant increase in cardiac contractility in the 8- and 12-week lead-treated rats (*P* < 0.05 and *P* < 0.01, respectively, as compared to controls) [Table 1]. No significant change was observed in coronary flow in any group Table 1.

There were no significant differences between control and 4-week lead-treated rats in any of the recorded parameters.

## Discussion

In the present study, subchronic exposure to low levels of lead increased HR and cardiac contractility as well as arterial blood pressure. This level of exposure over periods of 8-12 weeks prolonged the ST interval without causing any significant alteration in QRS duration. The incidence of arrhythmia and AV block increased only after 12 weeks of exposure, but there were no significant changes in coronary flow. Although it is possible that these findings cannot be extrapolated to humans, we used 100 ppm of lead acetate as the exposure level, which is considered as a low level of exposure and is similar to the levels seen in the environment; in contrast, other studies have examined the effects of higher (sometimes 50 times higher) industrial levels of exposure.[8,16] As has been reported earlier, 100 ppm of lead acetate in drinking water induced a rise in blood lead concentration in rats.[17] In our study, this amount of exposure increased the concentration of lead in the blood from the pre-exposure level of < 2 μg/dl to 26.84 ± 2.23 μg/dl after 12 weeks of exposure in adult rats. The increase in lead concentration in blood depends on both the dose of the metal and the duration of exposure.[18] Following the increase in plasma levels, the content of lead in the whole heart tissue (and other soft tissues) increases as well.[19]

In a previous experimental study, Boscolo and his colleague[20,21] demonstrated that exposure to 60 ppm of lead increases both blood pressure and cardiac inotropism, without causing any morphological changes in the myocardium. They confirmed that chronic lead exposure affects the cardiovascular system, not only by inducing metabolic changes (such as those related to the metabolism of Ca^+2^ or of the high-energy phosphate pathway) which are able to produce neurohumoral disorders, but also by increasing the neuorogenic and humoral effects that may be involved in the regulation of the metabolic processes. Moreover, it is also reported that systolic and diastolic blood pressure as well as cardiac positive inotropism (dp/dt) were increased by 50 or 100 ppm of lead. However, HR, ECG pattern, and negative dp/dt were not altered.[14,18] While, others have noted that HR increased dose-independently in lead-exposed rats,[4,15,19] we observed no certain relationship between tachycardia and duration of exposure. It seems that the HR remains unchanged in the early phases of exposure to low levels of lead, with tachycardia occurring only in the late stages. As the lead content of the body increases, the HR tends to decline because of lead-induced abnormalities in the cardiac conduction system. On the other hand, these observations are not consistent with a previous report of lead in the concentration of 10^−7^-10^−4^ M inducing a significant diminution in cardiac contractility in isolated frog hearts.[11] These differences are likely due to differences in experimental protocol such as the dose of lead, exposure procedure, and animal species.

Increased HR and contractility as well as arterial blood pressure in lead-exposed animals in our study may be due to the ability of this metal to alter cellular metabolism of Ca^+2^. Studies on Ca^+2^ and the vascular effects of lead have shown that intracellular Ca^+2^-binding sites are involved in the action of this metal in vascular smooth muscle.[19,20,22] A similar finding was reported in the case of the heart, as Ca^+2^ influx was observed in both atrial trabeculae and in the papillary muscles of the heart.[19] The increase in Ca^+2^ influx and elevation of intracellular content of Ca^+2^ by lead may be due to the action of this metal in altering Ca^+2^ transport processes, such as activation of protein kinase C (that activates Ca^+2^ channel opening),[23] inhibition of Na^+^ -K^+^, ATP_ase_,[11,24] and alteration in intracellular events.[18,23,25] The increased HR and cardiac contractility following the augmented intracellular Ca^+2^ concentrations causes hypertension due to increased cardiac output.[6] How far this increase in Ca^+2^ influx in cardiomyocytes contributes to the ECG changes in lead-exposed animals needs further investigations. As demonstrated in this study, low levels of lead in the early phases of exposure were not able to alter the rhythm of the heart, as arrhythmogenesis and AV block were elicited only in the late phases of exposure (after 12 weeks). So, the toxic effects of long-term, low-level lead exposure on the cardiac conducting system are more likely to be a chronic phenomenon. This possibility is consistent with other epidemiological studies in this regard.[10] Moreover, the decreased cardiac contractile function observed following chronic exposure to higher levels of lead may be attributable to the cardiac conduction defects which occur in that condition. Beside the Ca^2+^ pathway, other factors may also be involved in mediating the action of lead, eg, nitric oxide, catecholamines, endothelin, and the rennin-angiotensin system.[8,18,19]

It has been demonstrated that lead causes constriction of blood vessels through a direct effect on the endothelium and thus reduces blood flow;[22] however, this does not seem to be the case in our study, where the coronary flow did not differ significantly between control and lead-treated rats. It may be that lead tends to reduce coronary flow *per se* (by mechanisms such as inhibition of nitric oxide synthesis or increase of endothelin or rennin-angiotensin system activation) - we have previously reported that the relaxant effect of isoproterenol was significantly blunted in lead-treated groups as compared with control - but the coronary vessels are strongly under metabolic regulation. Therefore, following lead-induced increase in cardiac work, a metabolic vasodilatation occurs in coronary vessels and this factor actually prevents any coronary flow reduction.

In conclusion, the present findings demonstrate that subchronic exposure to low levels of lead induces an increase in HR and cardiac contractility as well as in arterial blood pressure. We found no certain relationship between tachycardia and duration of lead exposure. Cardiac conduction defects are less likely to occur in the early phases of exposure to low levels of lead: it seems to be the result of chronic exposure.

## References

[1] Jubery DR, Kleiman CF, Kwan SC (1997). Position paper of the American council on science and health: Lead and human health (review). Ecotoxicol Environ Safety.

[2] Kimberlie A, Charles VP (1997). Heavy metal toxicity; Part II: Lead and metal fume fever. J Emerg Med.

[3] Johnson FM (1998). The genetic effects of environmental lead. Mutat Res.

[4] Lai CC, Lin HH, Chen CW, Chen SH, Chiu TH (2002). Excitatory action of lead on rat sympathetic preganglionic neurons *in vitro* and *in vivo*. Life Sci.

[5] Needleman HL (1990). The future challenge of lead toxicity. Environ Health Persp.

[6] Pirkle JL, Schwartz J, Landis RJ, Harlan WR (1985). The relationship between blood lead levels and blood pressure and its cardiovascular risk implications. Am J Epidemiol.

[7] Perry MJ, Erlanger MW, Perry EF (1988). Increase in the blood pressure of rats chronically fed low levels of lead. Environ Health Persp.

[8] Karimi GR, Khoshbaten A, Abdollahi M, Sharifzadeh M, Namiranian K, Dehpour AR (2002). Effects of subacute lead acetate administration on nitric oxide and cyclooxigenase pathways in rat isolated aortic ring. Pharmacol Res.

[9] Heydari A, Khoshbaten A, Asgari AR, Noroozzadeh A, Ghasemi A, Najafi S (2006). Effects of short term subchronic lead poisoning on nitrice oxide metabolites and vascular responsiveness in rat. Toxicol Lett.

[10] Cheng Y, Schwartz J, Vokonas PS, Weiss ST, Avo A, Hu H (1998). Am J Cardiol.

[11] Krishnamoorthy MS, Muthu P, Parthiban N (1992). Effect of preperfusion of ascending concentrations of lead on digoxin-induced cardiac arrest in isolated frog heart. Toxicol Lett.

[12] Staessen J (1995). Low level lead exposure, renal function and blood pressure. Acad Geneskd Belg.

[13] Victery W (1988). Evidence for effects of chronic lead exrosure on blood pressure in experimental animals: An overview. Environ Health Persp.

[14] Carmignani M, Volpe AR, Boscolo P, Poma A, Boscolo P (1999). Kininergic system and arterial hypertension following chronic exposure to inorganic lead. Immunopharmacol.

[15] Evis MJ, Kane KA, Moore MR, Parratt JA (1985). The effects of chronic low lead treatment and hypertension on the severity of cardiac arrhythmias induced by coronary artery ligation in anesthetized rats. Toxicol App Pharmacol.

[16] Purdy RE, Smith JR, Ding Y, Oveisi F, Vaziri ND, Gonick HC (1997). Lead-induced hypertension is not associated with altered vascular reactivity in vitro. Am J Hypertens.

[17] Manton WI, Rothenberg SJ, Manalo M (2001). The lead content of blood serum. Environ Res Section A.

[18] Carmignani M, Volpe AR, Boscolo P, Qiao N, Gioacchino MD, Grilli A, Felaco M (2001). Catecholamine and nitric oxide systems as targets of chronic lead exposure in inducing selective functional impairment. Life Sci.

[19] Lal B, Murthy RC, Anand M, Chandra SV, Kumar R, Tripathi O (1991). Cardiotoxicity and hypertension in rats after oral lead exposure. Drug Chemical Toxicol.

[20] Boscolo P, Carmignani M (1988). Neurohumoral blood pressure regulation in lead exposure. Environ Health Persp.

[21] Boscolo P, Carmignani M (1988). Chronic low level lead exposure: Its role in the pathogenesis of hypertension. Med Toxicol.

[22] Chai SS, Webb RC (1988). Effect of lead on vascular reactivity. Environ Health Persp.

[23] Schanne FA, Long GJ, Rosen JF (1997). Lead induced rise in intracellular free calcium is mediated through activation of protein kinase C in osteoblastic bone cells. Biomechemica et Biophysica Acta.

[24] Weiler E, Khalil-Manesh F, Gonick H (1988). Effects of lead and natriuretic hormone on kinetics of sodium-potassium activated adenosine triphosphatase. Environ Health Persp.

[25] Goldstein GW (1993). Evidence that lead acts as a calcium substitute in second messenger. Metabolism.

